# Wild *Dictyostelium discoideum* social amoebae show plastic responses to the presence of nonrelatives during multicellular development

**DOI:** 10.1002/ece3.5924

**Published:** 2020-01-28

**Authors:** Suegene Noh, Lauren Christopher, Joan E. Strassmann, David C. Queller

**Affiliations:** ^1^ Department of Biology Colby College Waterville ME USA; ^2^ Department of Biology Washington University in St. Louis St. Louis MO USA

**Keywords:** microbial social evolution, plastic response, RNA‐seq, social conflict

## Abstract

When multiple strains of microbes form social groups, such as the multicellular fruiting bodies of *Dictyostelium discoideum*, conflict can arise regarding cell fate. Both fixed and plastic differences among strains can contribute to cell fate, and plastic responses may be particularly important if social environments frequently change. We used RNA‐sequencing and photographic time series analysis to detect possible conflict‐induced plastic differences between wild *D*. *discoideum* aggregates formed by single strains compared with mixed pairs of strains (chimeras). We found one hundred and two differentially expressed genes that were enriched for biological processes including cytoskeleton organization and cyclic AMP response (up‐regulated in chimeras), and DNA replication and cell cycle (down‐regulated in chimeras). In addition, our data indicate that in reference to a time series of multicellular development in the laboratory strain AX4, chimeras may be slightly behind clonal aggregates in their development. Finally, phenotypic analysis supported slower splitting of aggregates and a nonsignificant trend for larger group sizes in chimeras. The transcriptomic comparison and phenotypic analyses support discoordination among aggregate group members due to social conflict. These results are consistent with previously observed factors that affect cell fate decision in *D*. *discoideum* and provide evidence for plasticity in cAMP signaling and phenotypic coordination during development in response to social conflict in *D*. *discoideum* and similar microbial social groups.

## Graphical abstract

When multiple strains of microbes form social groups, such as the multicellular fruiting bodies of social amoebae, evolutionary conflict is expected to arise. We used RNA‐sequencing and photographic time series to detect possible conflict‐induced plastic differences between wild *Dictyostelium discoideum* aggregates formed by single strains compared with mixed pairs of strains (chimeras). Our results provide evidence for plastic responses in cell signaling and coordination that suggest amoebae can actively respond to the presence of nonrelatives during their single cell to multicellular developmental transition.

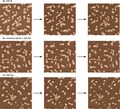

## 1 INTRODUCTION

Evolutionary conflicts of interest occur across many different types of cooperative alliances, including among cells within multicellular organisms (Burt & Trivers, 2006; Crespi & Summers, 2006). The facultatively multicellular amoeba *Dictyostelium discoideum* provides a convenient model to investigate conflict among cells of a social group in a multicellular context. *D*. *discoideum* becomes multicellular via aggregation rather than by cell division (Fisher, Cornwallis, & West, 2013; Grosberg & Strathmann, 2007). When soil‐dwelling amoebae starve, they produce a cyclic adenosine monophosphate (cAMP) signal and initiate a multicellular cycle in which tens of thousands of individual cells aggregate and ultimately develop into multicellular fruiting bodies (Kessin, 2001). Typical multicellular conflict control mechanisms such as single‐cell bottlenecks (Szathmáry & Maynard Smith, 1995) and early germ‐line sequestration (Buss, 1987) are potentially ineffective here, and the consequences of social conflict are either of two extremes. Amoebae that make it into the sorus (head) of a fruiting body become spores and survive, while the remainder will form the stalk and die during the transition to multicellularity (Strassmann & Queller, 2011; Strassmann, Zhu, & Queller, 2000). In response to low‐relatedness experimental evolution conditions in the laboratory, social amoebae readily evolve unfair representation as spores (cheating; Ennis, Dao, Pukatzki, & Kessin, 2000; Kuzdzal‐Fick, Fox, Strassmann, & Queller, 2011; Santorelli et al., 2008) and also counter‐evolve resistance to cheating (Hollis, 2012; Khare et al., 2009; Levin, Brock, Queller, & Strassmann, 2015).

The adaptive importance of social behaviors such as cheating in *D*. *discoideum* has been considered unclear for at least two reasons. First, studies have proposed that any apparent advantage of cheating in chimeras may be counterbalanced by life history and ecological trade‐offs. Some strains may produce more numerous but smaller and less viable spores relative to others (Wolf et al., 2015), and some strains may perform bet‐hedging against frequent environmental change by investing in more nonaggregating cells that can germinate more quickly when favorable conditions return (Martínez‐García & Tarnita, 2016; Tarnita, Washburne, Martinez‐Garcia, Sgro, & Levin, 2015). Second, *D*. *discoideum* possess highly polymorphic cell adhesion proteins (*tgrB1 and tgrC1* genes) that allow cells to bind better to others cells of their own strain under laboratory conditions, presumably for discrimination against nonrelatives (allorecognition) during fruiting body development (Benabentos et al., 2009; Gruenheit et al., 2017; Hirose, Benabentos, Ho, Kuspa, & Shaulsky, 2011). The presence of such loci indicates that mixed aggregations are not uncommon and perfect segregation via these *tgr* gene alleles would leave little opportunity for cheating, yet sorting is often incomplete. Spores within the same fruiting bodies, including those collected in the wild, can exhibit a range of relatedness (Flowers et al., 2010; Gilbert, Foster, Mehdiabadi, Strassmann, & Queller, 2007; Gilbert, Strassmann, & Queller, 2012; Ho & Shaulsky, 2015; Madgwick, Stewart, Belcher, Thompson, & Wolf, 2018; Ostrowski, Katoh, Shaulsky, Queller, & Strassmann, 2008).

Both fixed and inducible (plastic) differences among strains contribute to competition observed in the multicellular cycle (Buttery, Rozen, Wolf, & Thompson, 2009). The criticisms against whether these microbes display adaptive social behaviors tend to focus on fixed genetic differences that would lead to different competitive outcomes for strains that enter the multicellular cycle in mixes (Martínez‐García & Tarnita, 2016; Parkinson, Buttery, Wolf, & Thompson, 2011; Uchinomiya & Iwasa, 2013; Wolf et al., 2015). While plastic responses have the potential to be either adaptive or not, adaptive plastic responses should be selected for if social environments frequently change, for example, due to spore dispersal following a multicellular cycle. We used an RNA‐sequencing approach to identify potentially adaptive social plasticity genes that change expression specifically in response to chimerism and potential social conflict in multiple wild strains of social amoebae. We focused on the tight aggregate stage during the multicellular cycle, which marks the time point when aggregating single cells have just formed a compact multicellular body. At this stage, cell fate is being determined and gene expression patterns switch abruptly (Parikh et al., 2010; Rosengarten et al., 2015). Notably, among mutant strains, developmental arrests occur most often as tight aggregates rather than any other stage of development.

We compared patterns of gene expression at this stage under two social conditions: Clonal aggregates that contained only single wild strains versus chimeric aggregates in which two wild strains were mixed together in even proportion (1:1). We looked for genes that are expressed either more or less in chimeric aggregates as opposed to clonal aggregates, which we call chimera‐biased genes. These genes were previously reported to exhibit molecular signatures of rapid “arms‐race” adaptive evolution (Noh, Geist, Tian, Strassmann, & Queller, 2018), supporting the hypothesis that they are involved in conflict. Here, we explore the predicted functions of these differentially expressed genes in more detail and whether they can be related to specific phenotypic mechanisms of cheating.

We hypothesized that the expression patterns of chimera‐biased genes would be related to previously recognized factors known to affect cell fate of becoming stalk versus spore during multicellular development. These include cell cycle phase, and responsiveness to, and production of, cellular signals (Chattwood & Thompson, 2011; Gruenheit et al., 2018). We also examined expression patterns of previously reported candidate genes related to social behaviors in *D*. *discoideum*: Cheater genes had been identified by screening randomly mutagenized strains for genes that cause cheating when disrupted (Santorelli et al., 2008). If amoebae try to cheat in chimeras, and these genes cause cheating when disrupted, we hypothesized that chimeras may down‐regulate these genes. We also examined candidate genes that were up‐ and down‐regulated in a chimeric mixture of five genotypes (Hirose, Santhanam, Katoh‐Kurosawa, Shaulsky, & Kuspa, 2015). The amoeba strains mixed in this latter study were genetically engineered to differ only at their *tgrB1* and *tgrC1* loci on the axenic laboratory strain AX4 genetic background, in order to test whether these loci were primarily responsible for mediating multicellular development via discrimination using *TgrB1* and *TgrC1* for allorecognition.

We next tested whether our data were more consistent with either of two alternative hypotheses regarding how conflict may affect the progression of multicellular development in *D*. *discoideum*. We considered two previous hypotheses with opposite predictions: (a) cells in chimeras race to become spores (Kuzdzal‐Fick, Queller, & Strassmann, 2010) and (b) discoordination due to mixing two genotypes slows down development (Hirose et al., 2015). We compared the gene expression patterns of our samples to a developmental time series from the axenic laboratory strain AX4 (Rosengarten et al., 2015) and tested whether there was a consistent difference by social condition that may hint toward a relative acceleration or delay in development in chimeras.

Lastly, we investigated phenotypic effects of social conflict by analyzing photographic time series of development of the same strains of *D*. *discoideum* used in our RNA‐seq experiment. We expected to detect fixed phenotypic differences among strains but also to find plastic phenotypic responses to being in chimeras. We expected these phenotypic differences to be related to group size. For instance, aggregating amoebae of different genotypes have been hypothesized to form chimeras because larger slug sizes achieved in chimeras may allow for improved slug migration (Castillo, Switz, Foster, Queller, & Strassmann, 2005; Foster, Fortunato, Strassmann, & Queller, 2002). We hypothesized that if plastic responses allow chimeras to form larger aggregates, they may be adaptive based on models of social evolution in which an increase in group size also increases the chance of a cooperative outcome for social interactions (Peña, 2012).

## 2 METHODS

### 2.1 RNA‐sequencing and differential expression analysis

This experiment was previously described in Noh et al. (2018). Briefly, four pairs of *D*. *discoideum* strains, originally from Mt. Lake Biological Station in Virginia, were tested: *a* (QS6) with *b* (QS160), *c* (QS4) with *d* (QS174), *e* (QS18) with *f* (QS154), and *g* (QS17) with *h* (QS157). We grew amoebae from ~2 × 10^5^ spores per SM/5 plate (2 g glucose, 2 g BactoPeptone (Oxoid), 2 g yeast extract (Oxoid), 0.2 g MgCl_2_, 1.9 g KH_2_PO_4_, 1 g K_2_HPO_4_, and 15 g agar per liter) with 250 μl *Klebsiella pneumoniae* at 1.5 OD. We scraped *D*. *discoideum* cells in log‐phase growth from the agar plates and washed these cells three times with KK2 buffer (2.25 g KH_2_PO_4_, 0.67 g K_2_HPO_4_ in 1 L H_2_O). We then spread a total of 10^8^ cells suspended in 1 ml KK2 onto 47 mm diameter nitrocellulose filters (Millipore) moistened with KK2 to induce development. For each pair of strains, we prepared filters for the two unmixed clonal strains and for the 50:50 chimeric mix of strains, resulting in a trio of samples for each biological replicate pair (two clonal and one chimera; *e.g.* for pair ***ab*** *‐ a*, *b*, and *ab*). For each pair, we conducted three replicates of the experiment on different dates.

When ~90% of the filter area was covered with tight aggregates based on visual inspection, we washed off and resuspended cells into a 5× volume of RNAlater^®^ for storage at 4°C. Collecting at a fixed developmental stage by visual inspection resulted in chimeric samples being collected at a slightly earlier absolute time than exactly halfway between each of two clonal samples (Table 1). When all samples were collected, we extracted RNA using a protocol for cytoplasmic RNA purification from animal cells, with additional modifications based on Kaul and Eichinger 2006 with a Qiagen RNeasy^®^ Mini Kit and prepared sequencing libraries using the standard illumina protocol for the poly‐A‐tailed stranded mRNA library prep kit. We sequenced sample libraries on three lanes of an illumina Hiseq2500, for 50 bp single‐end reads at the Washington University in St. Louis Genome Technology Access Center (GTAC). The three replicates of the experiment were each done in a separate library preparation batch and sequenced on separate lanes. In other words, strains and treatments were balanced across batches and lanes. FASTQ files for all samples are available through NCBI Sequence Read Archive as Bioproject PRJNA526919.

**Table 1:** Timing of aggregation collection in minutes

Sample Pair	Replicate	Strain X	Chimera X + Y	Strain Y	Chimera relative time
QS6 + QS160	ab1	310	310	320	0.00
	ab2	300	285	285	0.00
QS4 + QS174	cd1	285	285	300	0.00
	cd2	270	270	240	1.00
	cd3	257	276	290	0.58
QS18 + QS154	ef1	332	292	302	−0.33
	ef2	312	297	280	0.53
	ef3	371	344	322	0.45
QS17 + QS157	gh1	240	248	260	0.40
	gh2	234	254	278	0.45
	gh3	273	280	286	0.54

Note

Chimera relative time measures how close the chimera (C) collection time as to the earlier (E) versus the later (L) of the two strains: ((C‐E)/(L‐E)) for example, 0 means the chimera was collected at the same times as the earlier strain, 1 at the same time as the later strain.

We aligned quality‐controlled reads (removed reads shorter than 12 bp and those with any N nucleotides) onto the *D*. *discoideum* reference genome (downloaded Dec 2014 from Ensembl Protist v1.25; chromosome 2 segmental duplication (2:3016083‐3768654) masked using bedtools v2.19.1 (Quinlan & Hall, 2010)) with GSNAP v2019‐06‐10 (Wu & Watanabe, 2005). We used Picard v2.17.10 (downloaded from broadinstitute.github.io/picard) to sort alignments and fix read groups, and collect alignment summary metrics. Next, we extracted read counts from uniquely mapped reads with correct strand orientation using HTSeq v0.11.2 (Anders, Pyl, & Huber, 2015). Subsequent analyses were run with R v3.6.0 (R Core Team 2019). At this point, we excluded one replicate of the strain pair *a* (QS6) and *b* (QS160) from our analyses because the reads were of overall low quality as reported by ShortRead v1.42.0 (Morgan et al., 2009). We used DESeq2 v1.24.0 (Love, Huber, & Anders, 2014) to test for evidence of significant differential expression and to determine which genes were differentially expressed. We tested 10,285 genes, filtered from the 12,451 genes with any coverage, by a threshold determined by DESeq2 (more than four reads per library). We used a GLM model (count ~ batch + pair + condition), with “batch” coding for the three sequencing lane and library preparation batches, “pair” coding for the four strain pairs used as biological replicates, and “condition” coding for the two social condition of aggregation, clonal versus chimeric. With this model, when the conditions are contrasted the two clonal samples within a strain pair will be considered together and compared to the chimeric sample.

### 2.2 Functional annotation and enrichment analyses

We used the R package GOstats v2.50.0 (Falcon & Gentleman, 2007) to test for significant overrepresentation of Gene Ontology (GO) terms in chimera‐biased genes compared to the universe of genes that were expressed among the samples. We first filtered a set of GO annotations (version 1 July 2018) from dictybase.org (Chisholm et al., 2006; Fey et al., 2009). Because of irrelevance or low reliability (Skunca, Altenhoff, & Dessimoz, 2012), we excluded annotations with “NOT” qualifiers, with the “ND (No biological Data available)” evidence code, or with “IEA (Inferred from Electronic Annotation)” evidence codes specifically from InterPro or HAMAP. While our enrichment tests included all available GO terms, we report results from GO terms whose members exceed 8 genes but are under 400 genes, because smaller terms can be subject to more false‐positive errors and larger terms are so broad that they are uninformative. Other considerations for using GO terms have been reviewed previously (Khatri & Drăghici, 2005; Rhee, Wood, Dolinski, & Draghici, 2008). GOstats uses conditional hypergeometric tests that take into account the nested structure of GO terms by testing child categories first. We also compared our gene lists to KEGG pathways using hypergeometric tests with FDR correction (0.05) in R. In addition to the GO enrichment analysis, we also estimated the overall expression patterns of the 196 GO Biological Process terms with 13 to 399 members with each gene occurring once at its deepest level in the GO hierarchy. For each of these GO terms, we found the annotated member genes in our differential expression results and calculated the median log2FC for each GO term.

With the RNA‐seq data, we also looked at the overall expression patterns of previously described sets of genes relevant to social competition in *D*. *discoideum*: cheater genes (Santorelli et al., 2008) and up‐ and down‐regulated genes from a 5‐way mixture of genotypes (Hirose et al., 2015). For cheater genes, we tested whether these genes were down‐regulated in chimeras relative to random expectation by comparing their mean expression against randomly selected sets of genes of the same size in a permutation test. We sampled log2 fold change (FC) values across our entire data set 10,000 times without replacement and tested how often the mean expression of a randomly selected set of genes was smaller than the log2FC observed for cheater genes. Hirose and colleagues compared clonal versus. 5‐way chimeric mixtures of engineered AX4 strains that differed only in their TgrB1 and TgrC1 alleles and looked at gene expression patterns from 8 and 12 hr into development. We specifically compared genes that this study found up‐ and down‐regulated in chimeras at 12 hr as this time point was the closest to tight aggregates according to their description. We had expression data from our experiment for 13 of the 14 up‐regulated genes and all 71 of the down‐regulated genes found by this study. We tested the up‐ and down‐regulation of these genes relative to random expectations using permutation tests as described above for cheater genes.

### 2.3 Inference of developmental progression by comparison to the reference strain AX4

To assess any gross differences in developmental progression in mixes, we compared our gene expression patterns with those from a study featuring gene expression at multiple time points during multicellular development of the laboratory strain AX4 (Rosengarten et al., 2015). We expect some differences in gene expression between AX4 and wild strains because AX4 is adapted to the laboratory environment, specifically to axenic liquid culture (Sussman & Sussman, 1967; Watts & Ashworth, 1970). Raw RNA‐seq reads were downloaded from NCBI Sequence Read Archive [BioProject SRP048533]. We specifically used the fastq files of the two replicates collected during filter development that were sampled at 19 time points from 00 to 24 hr. These samples had been prepared in a similar manner to our samples but were developed at half the cell density (5 × 10^7^ cells in 1,000 μl). We processed these raw data using the same pipeline as for our RNA‐seq reads. We regularized log (rlog) transformed the data in DESeq2 and generated a Euclidean distance matrix of pairwise comparisons between all AX4 time point samples and all clonal or chimeric samples from our experiment. The transformation was done while accounting for the differences in sequencing batches (experimental replicates in both experiments) by specifying “blind = FALSE” while using the rlog transformation function. We then averaged the Euclidean distances within each sample versus. each AX4 time point.

We calculated these pairwise distances between our samples and each of Rosengarten's time points in two different ways: once using all genes that were expressed in both experiments and a second time using only those genes that were reported as significantly differentially expressed by time (Rosengarten et al., 2015). We tried the second method in case it is more sensitive to any signal from the smaller number of genes that were informative for developmental progression. With both sets of distances, we tested whether social condition (clonal vs. chimeric) affected which AX4 time point each sample most resembled using generalized additive models with the R package mgcv v.1.8‐28 (Wood, 2006, 2011). Because the timing samples were not collected at even intervals, we used time point as a new continuous variable, referred to as “hr” below. For example, the first time point "00" was coded as “1,” an intermediate time point “11” was coded as “12” as it was the 12th time point collected, and the last time point “24” was coded as “19” as it was the 19th time point collected. We fitted the generalized additive models using restricted maximum likelihood (REML). The fitted models used thin plate regression splines to describe the relationship between distance and time, both with splines varying by social condition (distance ~ condition + s(hr, by = condition) + s(rep, bs = “re”)) and invariable by social condition (distance ~ condition + s(hr) + s(rep, bs = “re”)). For both models, we specified sample pair as a random effect. We selected the model with the lowest Akaike information criterion (AIC) score as the best model going forward if model fit was significantly better (Gurka, 2006). The local minima of the fitted splines (zero 1st derivative and positive 2nd derivative) would indicate which Rosengarten time point was most similar to our samples. We used finite differences to approximate the 1st and 2nd derivatives of the curve (Wood, 2006). This entailed using the fitted generalized additive model to predict the response variable at a specific step size (0.1) before and after the observed data points. We then approximated the 1st and 2nd derivatives by solving for the slope (1st derivative) and the change in the slope (2nd derivative) between these steps.

### 2.4 Phenotypic differences between strains in their multicellular development

**Figure 1:**
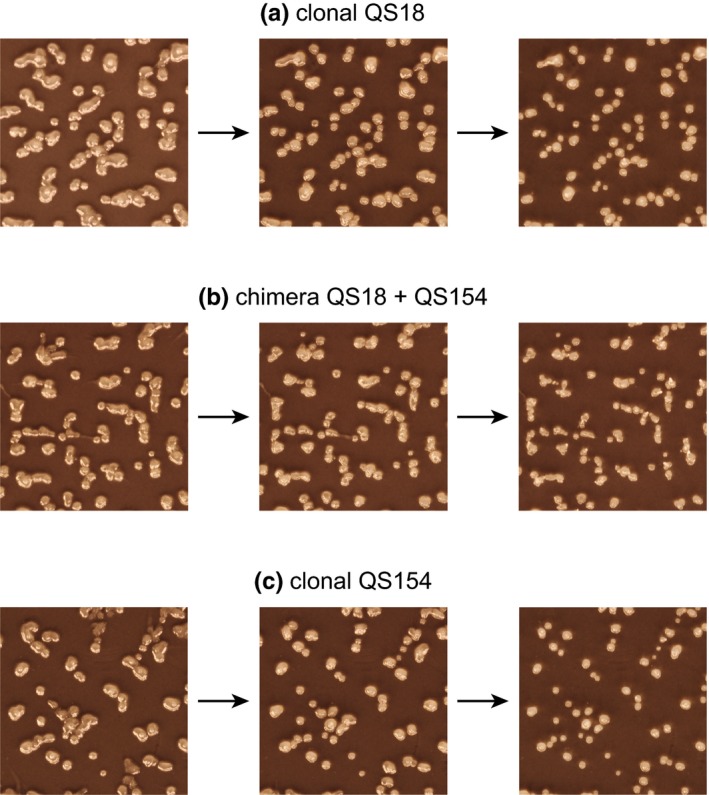
Partial fields of photographs analyzed for time series of phenotypic change during early development from one replicate pair **ef** (QS18, QS154). From left to right developing cells are in the form of loose, tight, and tipped aggregates

Lastly, we tested whether aggregate phenotype differed between the strains we used for RNA‐seq, both clonal and chimeric. We prepared cells in the same way as for the RNA‐seq experiment by growing them on bacterial lawns and collecting at log‐phase growth. After washing, we spread amoebae at 5 × 10^7^ cells per ml (in chimeras, 2.5 × 10^7^ cells per ml of each clone) onto premoistened nitrocellulose filters. We chose this density because the density that was used for the RNA‐seq experiment (1 × 10^8^ cells per ml) was too high for digital detection and scoring of separate aggregates using image analysis with the software ImageJ (Schindelin et al., 2012). We prepared one filter for each pair of strains and photographed developing filters at ~20‐min intervals using a digital camera fitted with a macro lens. All photographs were taken on the same photographic stage with the camera mounted at a fixed distance away from the platform on which the samples were placed. We determined that aggregates belonged to three developmental phases: loose aggregate (the aggregate no longer has streaming arms), tight aggregate (the edges of the aggregate are distinct from the substrate), and tipped aggregate (the aggregate has formed a nub that will eventually elongate into a slug during its mobile phase) (Figure 1). We used the function Particle Analyzer in ImageJ to automatically score aggregate size and shape parameters from these photographs. For the first time point, we analyzed between 50 and 198 aggregates (mean = 110.9) depending on the specific strain. For the last time point, we analyzed between 105 and 322 aggregates (mean = 184.6) per strain. We used custom Python code to track aggregates between photographs over time and determined whether an aggregate from one time point to the next split into smaller aggregates, merged into a larger aggregate, or remained intact.

We determined four shape parameters that had a pairwise correlation of less than 90% to describe the shapes of aggregates with the R package Schloerke et al., GGally v.1.4.0. (2011)These were area, circularity (=4π* (area/perimeter^2^); a value of 1 indicates a geometric circle), aspect ratio, and solidity (=area/(area of convex hull); a smaller value indicates a lobed or holey shape). We used principal components analyses to summarize shapes over time. We used the R package nlme v.3.1‐141 Pinheiro et al., 2019 to fit a mixed effects model to determine the combined effects of condition (clonal vs. chimeric) and time (loose aggregate, tight aggregate, and tipped aggregate) on aggregate shape (model: PC1 ~ condition:time + condition + time, random = ~1|strain). While the first mixed effects model was fit to 5,196 individual aggregates across three time points, we performed a second analysis on 2,865 aggregates from the first two time points for which we were able to track whether aggregates split into smaller ones over time. For this second analysis, we used a mixed effects model to determine the effect of aggregate shape (PC1) and the individual and combined effects of social condition and time interval on splitting (model: splitting ~ condition:time + condition + time + shape, random = ~1|strain). For both models, we specified strain identity as our random effect.

## 3 RESULTS

### 3.1 Differential expression between clonal versus chimeric development

**Figure 2:**
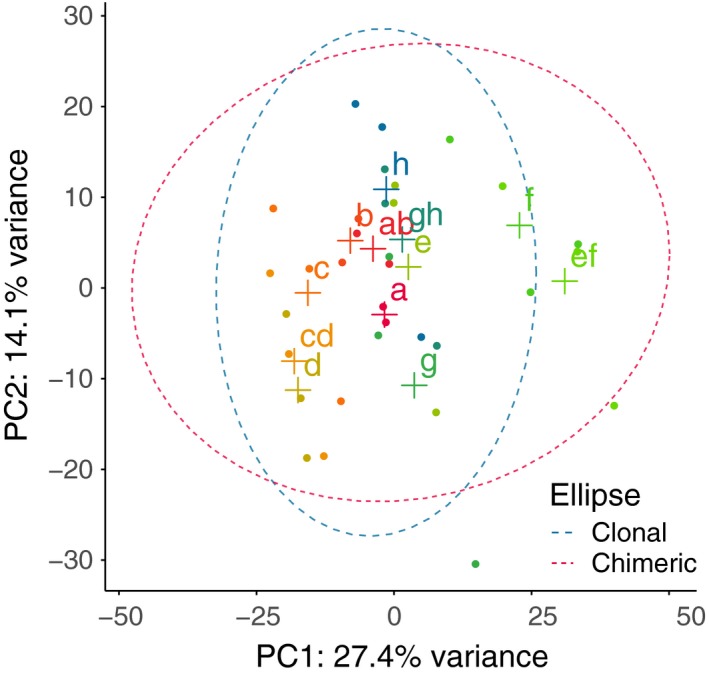
Overall gene expression patterns of clonal and chimeric tight aggregate samples from the current study. Samples tended to cluster by replicate pairs. Labels indicate the following strain identities throughout the supplement unless indicated otherwise: a (QS6); b (QS160); c (QS4); d (QS174); e (QS18); f (QS154); g (QS17); h (QS157)

**Figure 3:**
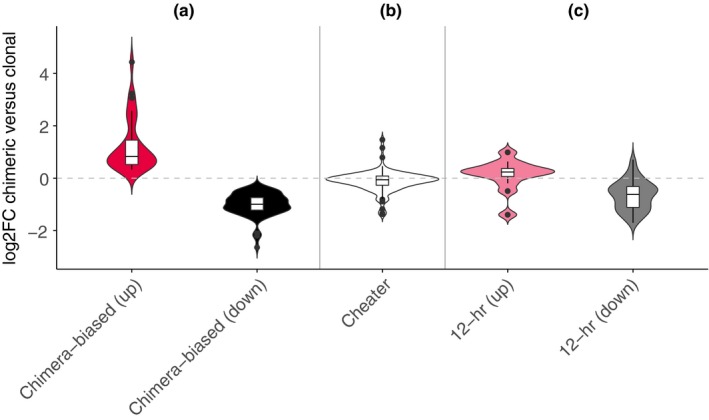
Expression patterns of 102 chimera‐biased genes (FDR < 0.1) shown in a heatmap. Individual samples are shown as columns, and genes in rows are clustered by expression pattern: over‐expressed in chimera (top and bottom) versus under‐expressed in chimera (middle)

**Figure 4:**
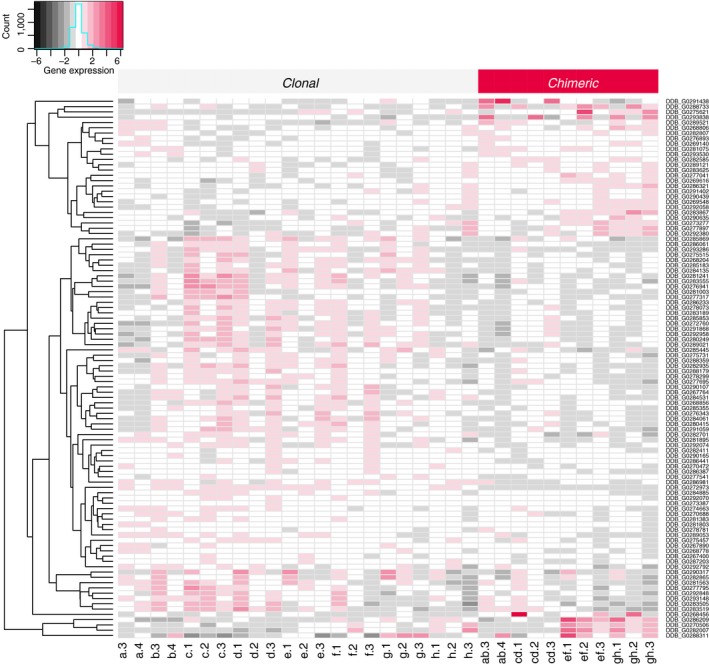
Expression patterns of sets of genes in mixed chimeras as opposed to clonal strains. We found (a) 31 significantly up‐regulated and 71 significantly down‐regulated chimera‐biased genes (FDR < 0.1); (b) no differential expression in previously identified cheater genes (Santorelli et al., 2008); and (c) consistent directional changes in previously identified up‐regulated or down‐regulated genes in a 5‐way mixture of genetically engineered genotypes differing only at kin recognition loci at 12 hr into development on filters (Hirose et al., 2015)

We identified inducible plastic responses to social conflict during the transition to multicellularity by contrasting chimeric expression against clonal expression of tight aggregates in *D*. *discoideum*. Expression patterns of clones and chimeras of the same strain pairs tended to cluster together, and chimeric patterns tended to be intermediate between the two contributing clonal patterns (Figure 2). We identified one hundred and two chimera‐biased genes in all four pairs of strains combined (FDR < 0.10). The fold change difference between clonal and chimeric conditions was relatively small across genes detected as chimera‐biased (Figure 3a, Figure 4). For those down‐regulated in chimeras (71 genes), the median fold change in expression was 0.50 (log2FC = −1.00 (*SE* 0.25)). For those up‐regulated in chimeras (31 genes), the median fold change in expression was 1.77 (log2FC = 0.83 (*SE* 0.22)).

**Figure 5:**
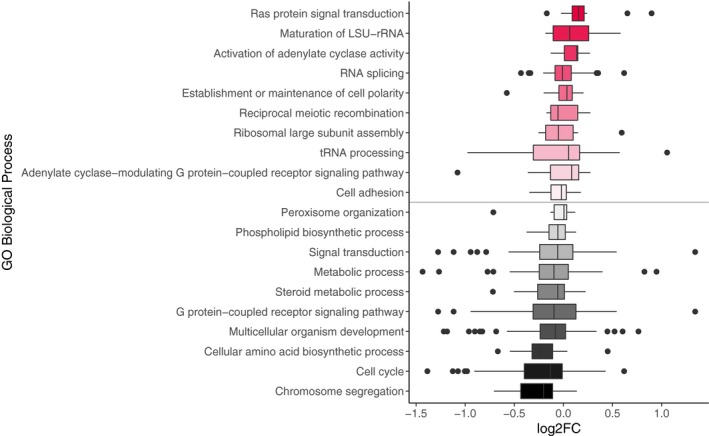
Expression patterns of groups of genes belonging to Gene Ontology Biological Processes. The top (red) sets of genes were most over‐expressed in chimeras, while the bottom (black) sets of genes were most under‐expressed in chimeras

Conditional tests of Gene Ontology (GO) term enrichment indicated that chimera‐biased genes that were significantly up‐regulated in chimeras were enriched for cytoskeleton organization and cAMP responses (Table 2; *p* < .05), and chimera‐biased genes that were significantly down‐regulated in chimeras were enriched for DNA replication and cell cycle (Table 3; *p* < .05). The KEGG pathway for DNA replication (ddi03030) was also significantly overrepresented in the down‐regulated chimera‐biased genes. When we looked at the expression patterns of groups of genes that made up major GO Biological Processes (196 terms), we found a median fold change of 0.96 (log2FoldChange = −0.06). In comparison, when we looked at the top 10 and bottom 10 terms, these overlapped with the results of the enrichment analyses for chimera‐biased genes (Figure 5).

**Table 2:** Gene Ontology terms enriched in chimera‐biased and up‐regulated set of genes

	Expected count	Observed count	Term size	*p*‐Value	Genes
Biological Process					
Regulation of myosin II filament assembly (GO:0043520)	0.044	2	13	.001	*ctxB*, *gapA*
Myosin filament assembly (GO:0031034)	0.064	2	19	.002	*ctxB*, *gapA*
Myosin II filament organization (GO:0031038)	0.088	2	26	.003	*ctxB*, *gapA*
Negative regulation of cellular component organization (GO:0051129)	0.189	2	56	.015	*gapA*, *rheb*
Response to drug (GO:0042493)	0.229	2	68	.021	*pde4*, *fhbB*
Regulation of actin cytoskeleton organization (GO:0032956)	0.239	2	71	.023	*ctxB*, *gapA*
Chemotaxis to cAMP (GO:0043327)	0.253	2	75	.025	*gapA*, *ctxB*
Negative regulation of phagocytosis (GO:0050765)	0.040	1	12	.040	*rheb*
Sexual reproduction (GO:0019953)	0.323	2	96	.040	*DDB_G0290635*, *gapA*
Regulation of cellular component biogenesis (GO:0044087)	0.327	2	97	.041	*ctxB*, *gapA*
Protein complex oligomerization (GO:0051259)	0.044	1	13	.043	*gapA*
Cyclic nucleotide metabolic process (GO:0009187)	0.051	1	15	.049	*pde4*
Molecular Function					
Protein‐lysine *N*‐methyltransferase activity (GO:0016279)	0.030	1	9	.030	*DDB_G0275621*
Rac GTPase binding (GO:0048365)	0.033	1	10	.033	*gapA*
Ubiquitin‐like protein‐specific protease activity (GO:0019783)	0.033	1	10	.033	*uch1*
Protein heterodimerization activity (GO:0046982)	0.043	1	13	.043	*pefB*
cAMP binding (GO:0030552)	0.043	1	13	.043	*pde4*

**Table 3:** Gene Ontology terms enriched in chimera‐biased and down‐regulated set of genes

	Expected count	Observed count	Term size	*p*‐Value	Genes
Biological process					
Positive regulation of gene expression (GO:0010628)	0.634	4	85	.003	*elof1*, *carB*, *mybC*, *sma*
Positive regulation of metabolic process (GO:0009893)	1.089	5	146	.004	*elof1*, *commd1*, *carB*, *mybC*, *sma*
Cell cycle (GO:0007049)	1.790	6	240	.008	*DDB_G0283189*, *mcm6*, *anapc6*, *ube2s*, *mcm5*, *DDB_G0280249*
DNA strand elongation (GO:0022616)	0.134	2	18	.008	*DDB_G0283189*, *rfc2*
DNA replication (GO:0006260)	0.197	2	29	.016	*mcm6*, *mcm5*
Regulation of proteolysis (GO:0030162)	0.231	2	31	.022	*DDB_G0286387*, *commd1*
DNA‐dependent DNA replication (GO:0006261)	0.296	2	41	.035	*DDB_G0283189*, *rfc2*
Protein modification by small protein conjugation (GO:0032446)	0.731	3	98	.035	*ube2s*, *commd1*, *anapc6*
Ubiquitin‐dependent protein catabolic process (GO:0006511)	0.828	3	111	.048	*DDB_G0286387*, *ube2s*, *commd1*
Molecular Function					
DNA binding (GO:0003677)	2.421	10	308	.000	*DDB_G0283189*, *polA4*, *DDB_G0290107*, *rfc2*, *mcm6*, *mybC*, *sma*, *bzpR*, *mcm5*, *cotA*
Dipeptidyl‐peptidase activity (GO:0008239)	0.071	2	9	.002	*DDB_G0274663*, *DDB_G0278299*
Serine‐type peptidase activity (GO:0008236)	0.299	3	38	.003	*DDB_G0268856*, *DDB_G0274663*, *DDB_G0278299*
Serine‐type carboxypeptidase activity (GO:0004185)	0.094	2	12	.004	*DDB_G0274663*, *DDB_G0278299*
Ubiquitin protein ligase binding (GO:0031625)	0.244	2	31	.024	*DDB_G0286387*, *ube2s*
Exopeptidase activity (GO:0008238)	0.338	2	43	.044	*DDB_G0274663*, *DDB_G0278299*
Cofactor binding (GO:0048037)	0.802	3	102	.045	*glpV*, *pah*, *DDB_G0283189*
Identical protein binding (GO:0042802)	0.346	2	44	.046	*asnB*, *pah*

We next checked the expression patterns of previously described sets of genes relevant to social competition. First, genes known to cause social cheating when knocked down (Santorelli et al., 2008) may be good candidates to be down‐regulated in chimeras, because this is the context in which cheating could be beneficial. However, these cheater genes were not significantly down‐regulated in chimeras any more so than random sets of genes (*p* = .26; Figure 3b). Second, to the extent that our changes were due to recognition, we expected genes that were previously shown to be up‐ and down‐regulated due to mismatch at kin recognition loci by Hirose et al. (2015) to show consistent directional changes in our data set. These predictions were satisfied (up‐regulated *p* = .03, down‐regulated *p* < .001), though interestingly the mean expression levels for these sets of genes were not as extreme as for the chimera‐biased genes we found (Figure 3c). We found no overlap between up‐regulated genes but 9 down‐regulated genes overlapped between the current study and that of Hirose and colleagues.

### 3.2 Inference of developmental progression by comparison to reference strain AX4

**Figure 6:**
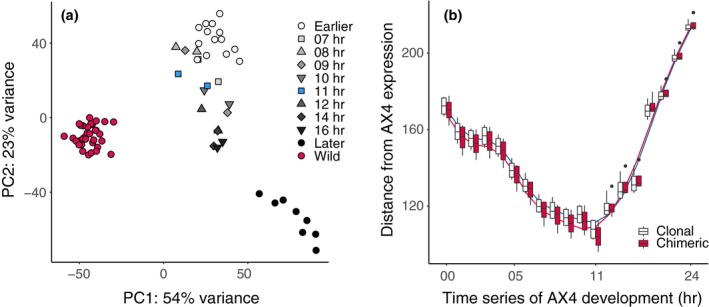
Comparison of data from current experiment to developmental time series of laboratory strain AX4 (Rosengarten et al., 2015). (a) Wild strains of *D*. *discoideum* used in this study (both clonal and chimeric) show difference in overall transcriptomic profile compared to AX4. (b) Clonal and chimeric samples are most similar to AX4 at the 11‐hr time point when compared across genes that change expression during development, but the fitted generalized additive models (GAM) splines indicate that the local minimum of the clonal model is closer to 11‐hr while chimeric is closer to 10‐hr

In order to test for an effect of conflict on developmental progression, we compared the expression patterns of our tight aggregate samples to those from a developmental time series of the *D*. *discoideum* reference laboratory strain AX4 (Rosengarten et al., 2015). Our data were most similar to expression patterns from 11 hr, thus matching the approximate morphological developmental stage (tight aggregate) our samples corresponded to (Figure 6a). When we tested whether social condition (clonal vs. chimeric) affects the progression of development differently, we found some evidence to support different rates of development depending on social condition when considering only those genes that showed differential expression during development (ΔAICc = 1.72, deviance = 1549.6, *df* = 9.12, *p* = .03). When we used all genes expressed across experiments, the difference between models was not significant (ΔAICc = 7.87, deviance = 1,134.3, *df* = 8.81, *p* = .50). What follows are the results of the comparison using developmental timing genes. The fitted generalized additive model explained 91.7% of the deviance present in the data (REML = −4576.7), and the residuals did not show nonrandom patterns that would indicate inadequately captured variance with our model. The different fitted splines of the generalized additive model indicated that the local minimum for clonal samples was closer to the 11‐hr time point, while the local minimum for chimeric samples was closer to the 10‐hr time point. Curiously, chimeras resembled AX4 significantly more than clonal samples based on the parametric coefficient estimate for social condition from the fitted model (*t* = −3.07, *p* = .002) (Figure 6b).

### 3.3 Phenotypic differences between strains in their multicellular development

**Figure 7:**
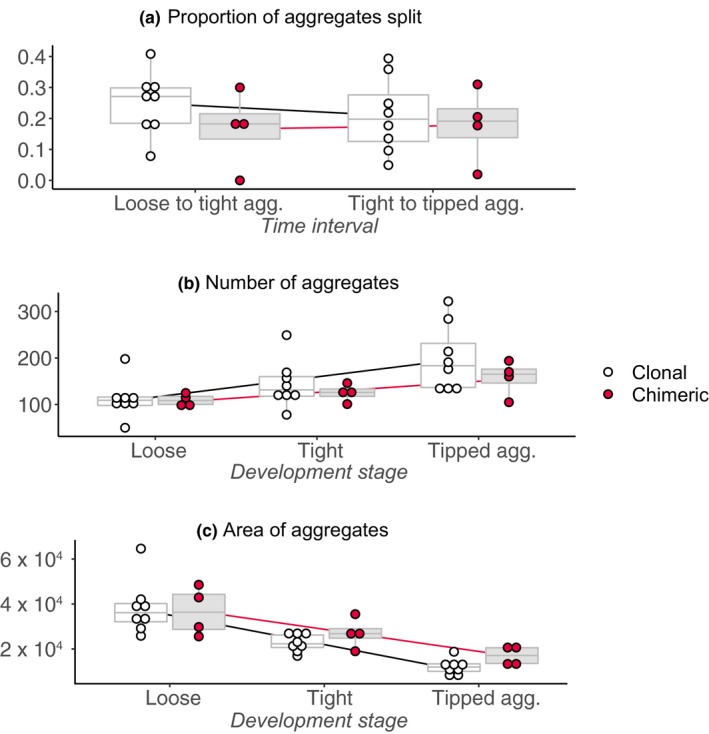
Photographic time series analysis of phenotypic change during early development. (a) While both clonal (left in each comparison) and chimeric (right) aggregates split into smaller aggregates during early development they do so following different patterns, with chimeras splitting at a lower rate than clonal (*F*_condition:time_ = 6.52, *df* = 1, *p* = .01). This results in (b) slightly fewer but (c) not significantly larger aggregates forming in chimera

Each strain of wild *D*. *discoideum* initially showed different overall shapes of aggregates during the transition to multicellular fruiting bodies. However, larger aggregates generally split into smaller ones over the course of development, and aggregate shapes across all strains converged over time into small circular aggregates that would subsequently elongate into motile slugs (Figure 7). While the difference in shape (PC1) between clonal and chimeric samples was not significant (*F*_condition_ = 3.98, *df* = 1, *p* = .07), the rate at which aggregate shapes changed in chimeric samples was significantly slower compared with clonal samples (*F*_condition:time_ = 73.84, *df* = 2, *p* < .001).

The rate at which aggregates split over the course of development in clonal samples decreased over time while chimeric samples split at the same rate across time intervals (*F*_condition:time_ = 6.52, *df* = 1, *p* = .01) (Figure 7a). Aggregates overall split more often during the earlier interval compared with the later interval (*F*_time_ = 7.45, *df* = 1, *p* = .006), and shape affected whether an aggregate would split (*F*_PC1_ = 2093.67, *df* = 1, *p* < .001). The net effect is that while clonal and chimeric samples initially form roughly the same numbers of aggregates, chimeric samples ultimately end up with slightly fewer but larger aggregates (Figure 7b,c). The mean area of chimeric aggregates just prior to the slug stage was about 12,000 while clonal aggregates were about 7,000. The confidence limits overlapped because of the large variance in aggregate size and limited sample size; therefore, this difference was not statistically significant.

## 4 DISCUSSION

Apparent social conflict among unrelated strains of *D*. *discoideum* has previously been observed in laboratory conditions (Hilson, Kolmes, & Nellis, 1994; Hollis, 2012; Khare et al., 2009; Kuzdzal‐Fick et al., 2011; Levin et al., 2015; Strassmann et al., 2000), but the difference between fixed versus. plastic responses to social conflict has not received as much deliberate attention (Buttery et al., 2009). We combined pairs of wild *D*. *discoideum* strains and compared clonal and chimeric gene expression profiles at the specific stage in which the transition from unicellularity to multicellularity occurs. We found genes that were chimera‐biased in expression that point to potential adaptations specific to social conflict in *D*. *discoideum*. We investigated whether any further information regarding these potential adaptations can be obtained from the predicted functions of chimera‐biased genes, or from phenotypic comparisons of chimeric and clonal aggregates. Gene expression differences between chimeric and clonal development need not be adaptive. However, our chimera‐biased candidate genes invoke enriched GO categories and specific functions that are consistent with two known factors that influence cell fate: cyclic AMP signaling and cell cycle.

### 4.1 Chimerism and cAMP signaling

Multicellular development in *D*. *discoideum* is affected by how amoebae produce and degrade cAMP, as well as how they detect and relay its signal (Kessin, 2001; Loomis, 2015). Previous studies have shown that cells that start the cyclic AMP signal relay are more likely to become spores (Huang, Takagawa, Weeks, & Pears, 1997; Kuzdzal‐Fick et al., 2010). Models have shown that fixed differences among strains in production and sensitivity to diffusible social signals can lead to appearances of social cheating that resemble experimental results when in fact no social interactions are factored into the model itself (Martínez‐García & Tarnita, 2016; Parkinson et al., 2011; Uchinomiya & Iwasa, 2013). However, there is some evidence that some of these cAMP phenotypes can be phenotypically plastic depending on social context. For example, cells that are not physically connected to aggregate mounds during aggregation are significantly less responsive to cAMP compared to cells of the same strain that were inside mounds (Hirose et al., 2015).

**Figure 8:**
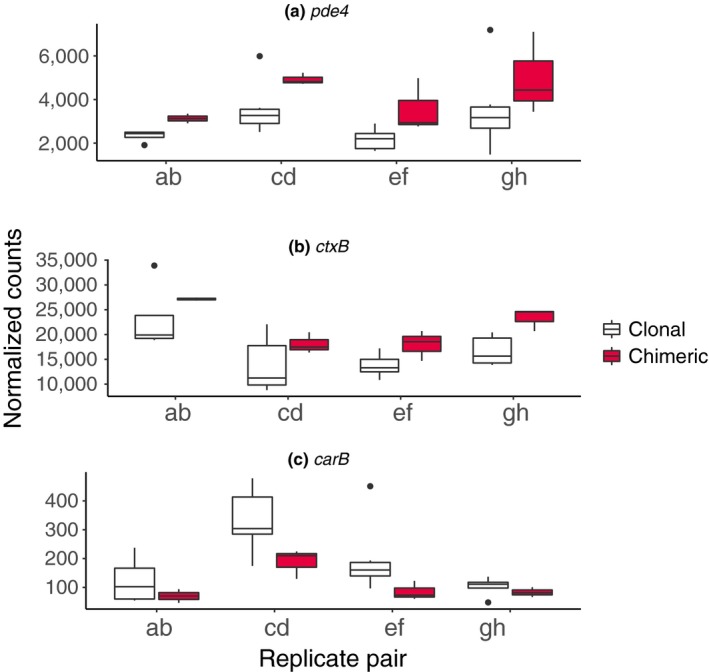
Examples of induced cAMP signaling‐related gene expression changes in response to chimeric condition during aggregation in wild strains of social amoebas include (a, b) up‐regulation of *pde4* and *ctxB* and (c) down‐regulation of *carB*

Our results support that *D*. *discoideum* strains can adjust cytoskeleton organization and cAMP signaling in response to potential social conflict (Table 2, Figure 8). We found two cAMP signal relay genes, *pde4* and *ctxB*, that are over‐expressed in chimeras. Pde4 is one of three membrane‐bound cAMP‐specific phosphodiesterases in *D*. *discoideum* and creates a local cAMP gradient that enables chemotaxis during multicellular development (Bader, Kortholt, Snippe, & Haastert, 2006; Bader, Kortholt, & Haastert, 2007). CtxB is an actin‐bundling protein also known as cortexilin II, which dimerizes with cortexilin I to enable aggregating groups of amoebae to physically respond to extracellular cAMP during the multicellular cycle (Shu, Liu, Kriebel, Daniels, & Korn, 2012). A third cAMP signal relay gene *carB* was down‐regulated in a chimera‐biased manner. CarB is a low‐affinity cAMP receptor that is preferentially expressed in cells with prestalk fate in tight aggregates (Dormann, Kim, Devreotes, & Weijer, 2001; Kim, Borleis, & Devreotes, 1998; Saxe et al., 1993).

### 4.2 Chimerism and cell cycle

Recent advances in our understanding of cell fate decision in *D*. *discoideum* show that many of the previously recognized factors that affect spore versus stalk fate (Chattwood & Thompson, 2011) can be traced back to stochastic, nongenetic heterogeneity among populations of cells related to cell cycle phase. For example, it now appears that vegetative cells within the same strain show stochastic variation in cell cycle phase in proportions that roughly correspond to future cell fate were the population of cells to enter multicellular development (Gruenheit et al., 2018). The same study shows how cell cycle phase is linked to glucose availability and DIF‐1 signaling. DIF‐1 and cAMP are known antagonists during *D*. *discoideum* development (Sugden, Urbaniak, Araki, & Williams, 2015).

Our results indicate that amoebae developing in chimeras down‐regulated DNA replication and cell cycle‐related genes. As noted above, more recently divided cells are more likely to become stalk rather than spores (Araki, Nakao, Takeuchi, & Maeda, 1994; Chattwood & Thompson, 2011; Gomer & Firtel, 1987; Thompson & Kay, 2000; Wood et al., 1996). Although social amoebae can undergo mitosis during the multicellular cycle, this appears to be rare (Muramoto & Chubb, 2008) and cell cycles are controlled by repressors of proliferation (but not growth) such as AprA, CfaD, and PakD during the multicellular cycle (Brock & Gomer, 2005; Gomer, Jang, & Brazill, 2011; Phillips & Gomer, 2014). Additional work is necessary to determine whether the plastic responses to chimerism we observed in down‐regulated DNA replication and cell cycle‐related genes are part of a signaling cascade (Gruenheit et al., 2018) that affects cell fate rather than directly affecting cell division itself.

### 4.3 Chimerism and cheating

In comparison, previously identified genes related to social behavior in *D*. *discoideum* did not respond to chimerism as expected (Figure 3b). These cheater genes, which cause cheating when knocked down (Santorelli et al., 2008), were not relatively down‐regulated in chimeras. We did confirm consistent directional expression changes of candidate genes identified from a 5‐way chimeric mix of strains that were engineered to differ only at allorecognition loci (Hirose et al., 2015) in our data (Figure 3c), although we found little overlap between these candidate genes and our significantly differentially expressed genes. This latter result suggests to us that while allorecognition loci are important for social interactions during the multicellular cycle (Gruenheit et al., 2017; Hirose et al., 2015), they are unlikely to be the exclusive factor that will determine the fate of these interactions. The former result may simply be due to the large insertions that generated the cheater mutants acting at different developmental stages than the one we examined. However, while this paper was in review a molecular evolution study of four overlapping sets of social genes in *D*. *discoideum* was published and it provides an alternative explanation. Oliveira et al. (2019) distinguished sociality genes (genes expressed during the multicellular cycle), chimerism genes (genes up‐regulated in chimeras relative to clonal aggregations at a later stage than our study, the slug stage), antagonism genes (genes differentially expressed between prespore and prestalk cells during development; Parikh et al., 2010), and cheater genes (as above; Santorelli et al., 2008). Their observation of little overlap in membership between sociality, chimerism, and cheater genes is also consistent with our results and a reviewer's observation that the recognition of nonrelatives and cheating may be physiologically distinct behaviors under the influence of largely different sets of genes.

### 4.4 Chimerism and phenotypic coordination

Given the adaptations to social conflict suggested by our transcriptomic results, we tested how this conflict may affect developmental progression and phenotypic change in aggregate shape and size. Both the transcriptomic comparison and phenotypic analyses support discoordination among aggregate group members due to social conflict. In our experiment, chimeric samples collected at the same phenotypic developmental stage showed signs of progressing through development in a significantly different form, and local minima of fitted generalized additive model splines comparing gene expression patterns indicated that chimeras may be slightly behind clonal samples (Figure 6; Table 1). We interpret these results with caution because of the striking difference in gene expression between our wild strains compared to the time series from the laboratory strain AX4, and how chimeras were relatively more similar to AX4. This may indicate that chimeras are regulating gene expression in a direction that corresponds with the direction that AX4 has evolved in the laboratory, under conditions that inadvertently select for cheaters (Kuzdzal‐Fick et al., 2011; Santorelli et al., 2008). The relatedness structure faced by any new cheater mutants that arise will determine how likely cheaters are able to persist: are they clustered with other cheaters (high relatedness) or well mixed with cooperator strains (low relatedness). Previous experiments have confirmed that liquid media culture conditions under which AX4 is often propagated create low‐relatedness conditions and selects for cheater mutations (Kuzdzal‐Fick et al., 2011).

In addition, our phenotypic results suggest that chimeras have less frequent aggregate splitting and a tendency toward increased group sizes (Figure 7). Variation in rates of splitting indicates plasticity in coordination among cells during multicellular development in *D*. *discoideum* as was previously found (Gruenheit et al., 2017; Hirose et al., 2015). Though our data are suggestive of larger group sizes in chimeras, the difference was not statistically significant and warrant replication with a larger number of wild strains. Slower rates of splitting in chimeras could be the result of discoordination due to incompatibilities in cellular signals that arose between strains evolving in isolation, analogous to Dobzhansky‐Muller incompatibilities (Rendueles et al., 2015). Conversely, increased group sizes could be adaptive based on kin selection if a given genotype becomes more likely to find other closely related individuals within a group as group size increases (Biernaskie & West, 2015). The effect of group size on the evolution of cooperation has often been overlooked but has important implications (Archetti, 2009; Garcia & Monte, 2013; Peña, 2012). For example, when group size is variable, cooperation evolves more readily compared to when group size is fixed (Peña, 2012), provided that the individual cost to benefit ratio is low (i.e., relative benefit is high). Our phenotypic results highlight a potentially important gap in knowledge in experimental models of social interactions in *D*. *discoideum* and other similar microbial social groups.

As pointed out by a reviewer, a caveat of the differential expression analysis we performed here is that subtler forms of transcriptomic plasticity would not be detected. For example, we would not be able to detect genotype‐specific gene expression where the responses of one genotype cancel out the responses of the partner. This possibility is something that we are actively pursuing, more readily now with the increasingly available bioinformatic tools that are built specifically to work with RNA‐sequencing data in an allele‐aware manner (Castel, Levy‐Moonshine, Mohammadi, Banks, & Lappalainen, 2015; Pirinen et al., 2015). But at the same time, we emphasize that our current approach in employing a standard GLM‐based differential expression analysis is that the chimera‐biased genes we found are likely to be a relatively robust sampling of genes that would be differentially expressed when a random pair of wild genotypes come together in a chimeric aggregate.

To conclude, our results support that plasticity in cell signaling and group size should be considered alongside variation in fixed factors such as allorecognition type (Gruenheit et al., 2017; Hirose et al., 2015), cell or stalk size (Votaw & Ostrowski, 2017; Wolf et al., 2015), and bet‐hedging by means of nonaggregating cells (Martínez‐García & Tarnita, 2016), to fully understand how social conflict affects the evolution of aggregative social groups in *D*. *discoideum* and other microbes. At a crucial developmental stage during the multicellular cycle of social amoebae when the transition from unicellularity to multicellularity occurs, we observed up‐regulation of cAMP signaling‐related genes and down‐regulation of cell cycle‐related genes when amoebae enter the multicellular cycle in chimeric mixes. Patterns of phenotypic change during development among *D*. *discoideum* strains indicate that increased conflict in chimeric mixes may result in larger group sizes formed by decreased coordination in aggregate splitting. Plasticity in cAMP signaling, cell cycle‐related processes, and coordination during the multicellular cycle, combined with evidence from molecular evolutionary patterns (Noh et al., 2018), support that social conflict with nonrelatives is a significant aspect of evolution in these microbes.

## CONFLICT OF INTEREST

The authors declare that we have no competing interests related to this work.

## AUTHOR CONTRIBUTIONS

SN, JES, and DCQ conceived and designed this study. SN and LC acquired the data. SN, LC, JES, and DCQ analyzed and interpreted the data, and drafted the manuscript. SN revised the manuscript.

## Data Availability

FASTQ raw sequence data files for all samples are available through NCBI Sequence Read Archive as BioProject PRJNA526919. All input data for both genomic and phenotypic analyses, output data from the genomic analyses, and all R code necessary for recreating the analyses in this paper are deposited to Dryad at the following URL: https://doi.org/10.5061/dryad.tqjq2bvtv
