# Corrigendum

**DOI:** 10.1002/ece3.9422

**Published:** 2022-10-11

In the recent article by Noh et al. (2020), the captions for Figures 3 and 4 were switched in the published version.

The correct captions are shown below:

FIGURE 3 Expression patterns of sets of genes in mixed chimeras as opposed to clonal strains. We found (a) 31 significantly up‐regulated and 71 significantly down‐regulated chimera‐biased genes (FDR < 0.1); (b) no differential expression in previously identified cheater genes (Santorelli et al., 2008); and (c) consistent directional changes in previously identified up‐regulated or down‐regulated genes in a 5‐way mixture of genetically engineered genotypes differing only at kin recognition loci at 12 h into development on filters (Hirose et al., 2015).

FIGURE 4 Expression patterns of 102 chimera‐biased genes (FDR < 0.1) shown in a heatmap. Individual samples are shown as columns, and genes in rows are clustered by expression pattern: overexpressed in chimera (top and bottom) versus under‐expressed in chimera (middle).

The authors apologize for the errors.
